# Prediction of adverse pregnancy outcomes using uterine artery Doppler imaging at 22-24 weeks of pregnancy: A North Indian experience

**DOI:** 10.4274/tjod.55632

**Published:** 2016-06-15

**Authors:** Deepti Verma, Sangeeta Gupta

**Affiliations:** 1 Maulana Azad Medical College, Department of Obstetrics and Gynecology, New Delhi, India

**Keywords:** Uterine artery Doppler, preeclampsia, fetal growth restriction, low birth weight, adverse obstetric outcome

## Abstract

**Objective:**

The aim of this study was to assess the predictive value of uterine artery Doppler imaging at 22-24 weeks of gestation for adverse pregnancy outcomes.

**Materials and Methods:**

This was a prospective study in which uterine artery Doppler was performed at 22-24 weeks of gestation in 165 pregnant women with singleton pregnancies. A pulsatility index (PI) more than 1.45 or bilateral uterine notching was labeled as abnormal Doppler. The pregnancy outcome was assessed in terms of normal outcome, preeclampsia, fetal growth restriction (FGR), low birth weight, spontaneous preterm delivery, oligohydramnios, fetal loss or at least one adverse outcome.

**Results:**

Out of 165 patients, 35 (21.2%) had abnormal second trimester uterine artery Doppler. In pregnancies that resulted in preeclampsia (PE), (n=21), FGR, (n=21), and low birth weight (n=39), the median uterine artery PI was higher (1.52, 1.41, and 1.27 respectively). In the presence of abnormal Doppler, the risk of PE [OR=10.7, 95% confidence interval (CI): (3.91-29.1); p<0.001], FGR [OR=4.34, 95% CI: (1.62-11.6); p=0.002], low birth weight [OR=6.39, 95% CI: (3.16-12.9); p<0.001] and the risk of at least one obstetric complication [OR=8.73, 95% CI: (3.5-21.3); p<0.001] was significantly high. The positive predictive value of abnormal uterine artery Doppler was highest for preeclampsia (36.84%) among all adverse pregnancy outcomes assessed.

**Conclusion:**

Uterine artery Doppler ultrasonography at 22-24 weeks of gestation is a significant predictor of at least one adverse pregnancy outcome, with the highest prediction for preeclampsia.

## INTRODUCTION

Defective trophoblastic invasion of the spiral arteries is associated with subsequent development of preeclampsia, fetal growth restriction (FGR), and other associated complications. In these pregnancies, the uteroplacental circulation remains in a state of high resistance, which causes generalized endothelial cell injury, compromises vascular integrity, and an atherosis-like process in small arteries that results in vascular occlusion, local ischemia, and necrosis. Under these conditions, the uteroplacental circulation remains in a state of high resistance and low flow. Doppler ultrasonography is a noninvasive tool for evaluating vascular resistance in these otherwise inaccessible maternal vessels. An increase in uterine artery impedance that predates the onset of clinical findings has been shown in preeclampsia and FGR; both disorders are associated with placental insufficiency^(1)^.

Uterine artery Doppler is an attractive screening test in mid pregnancy. In theory, the test is capable of identifying pregnancies that are at premature delivery from a range of clinical complications that are attributable to chronic placental disease, namely intrauterine growth retardation, abruption and preeclampsia. In this study, uterine artery Doppler was done in 20-24 weeks of gestation, contrary to the first trimester and early second trimester in many studies done previously^(1,2,3)^. The timing was based on the hypothesis that it is unlikely that the fetal growth and well being are influenced by the transformation of uteroplacental vessels at 11 weeks to 13 weeks 6 days period of gestation because substantial changes in the placental development take place between first and second trimester that have practical importance for the development of the clinical screening program^(4)^.

Uterine artery Doppler velocimetry performed before 16 weeks of gestation is unlikely to be a useful screening test for adverse pregnancy outcomes^(5)^. Moreover, it is irrational to employ this test for pre-eclampsia in the third trimester because the disease would already be established^(6)^.

This study was performed to assess the role of second trimester uterine artery Doppler in predicting adverse pregnancy outcomes in pregnant women in Northern India.

## MATERIALS AND METHODS

**Figure 1:**
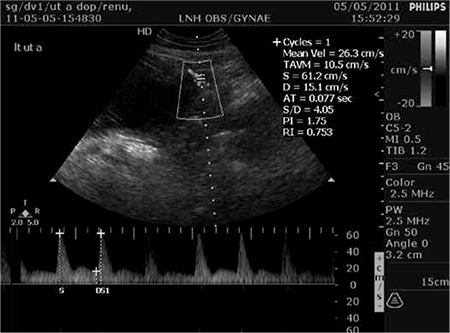
Second trimester uterine artery Doppler showing notching in left uterine artery and pulsatility index >1.45

This was a prospective study including 165 pregnant women with singleton pregnancies who attended the antenatal outpatient department of the Maulana Azad Medical College, Department of Obstetrics and Gynecology, New Delhi, India. Ethical committee approval was taken from institutional Ethics Committee, Maulana Azad Medical College, New Delhi (No: F.11/IEC/MAMC/10) and all subjects gave informed consent to participate in the study. Women with insulin-dependent diabetes mellitus, chronic hypertension, hypertension that developed before 24 weeks of pregnancy, cardiac disorders, renal disorders and antiphospholipid syndrome, and multiple gestations were excluded. A detailed informed consent was obtained from all participants after enrolment in the study. Uterine artery Doppler was performed in all these women at 22-24 weeks of gestation using a Philips HD7 ultrasound machine (number-Ci52100333). Uterine artery Doppler was performed by a single observer using a 225-Hz transabdominal probe. The proximal uterine arteries were located at their cross-over point with the external iliac arteries using color flow mapping. The angle of insonation was zero to ten degrees. Pulsed-wave Doppler was obtained for 3 similar consecutive waveforms on both sides. The pulsatility index (PI) was calculated [peak systolic flow minus end diastolic flow divided by mean flow: (A-B)/M]. Women with bilateral uterine artery notches or those with the mean PI of both the arteries ≥1.45 (PI greater than 95^th^ percentile) were classified as abnormal second trimester uterine artery Doppler (Figure 1).

Follow-up continued till delivery and the pregnancy outcome was assessed. Pregnancy outcome was assessed in terms of normal outcome, preeclampsia, FGR, low birth weight (weight <2500 gm), spontaneous preterm delivery, oligohydramnios, fetal loss, or at least one adverse outcome.

The statistical analysis of the data was performed using Statistical Package for the Social Sciences (SPSS) statistical software (SPSS Inc., Chicago, IL, version 17.0 for Windows). Qualitative data was analyzed using chi-square or Fisher’s exact test. Quantitative data between groups were analyzed using unpaired t-test and Mann-Whitney U test. A p value less than 0.05 was considered significant.

## RESULTS

The results are summarized in Table 1. Out of 165 patients, 35 (21.2%) had abnormal second trimester uterine artery Doppler, based on the criteria mentioned before (PI≥1.45 or bilateral uterine artery notches or both). The results indicated that second-trimester uterine artery Doppler significantly determined adverse obstetric outcomes. The incidence of preeclampsia, FGR, small-for-gestational-age neonates, and oligohydramnios was significantly higher in pregnant women with abnormal second-trimester uterine artery Doppler. Second-trimester uterine artery Doppler had high negative predictive value for preeclampsia in this study. No significant association was found between abnormal second-trimester uterine artery Doppler and incidence of preterm delivery in patients in the study group.

One case each of placental abruption and intrauterine fetal death was noted in the study group with abnormal second-trimester uterine artery Doppler.

Abnormal second-trimester uterine artery Doppler was found to have a high predictive value for at least one adverse obstetric outcome. At least one adverse obstetric outcome was found in 84.3% patients with abnormal Doppler indices. Mean uterine artery PI was significantly raised in these patients with a high and statistically significant odds ratio.

**Figure 2:**
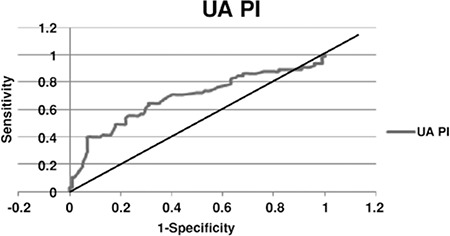
Receiver operator characterstics curve for uterine artery Doppler in depicting composite adverse pregnancy outcomes in the whole study group *The receiver operator characterstics curve for uterine artery Doppler in the whole study group revealed that the second trimester uterine artery Doppler is a significant predictor of adverse pregnancy outcomes. The area under the curve (AUC) is 0.659, 95% CI=0.562-0.756, p=0.001* *PI: Pulsatility index, UA: Umbilical arterial*

The receiver operator characterstics curve for uterine artery Doppler in the detection of composite adverse outcomes in the whole study group revealed that second-trimester uterine artery Doppler was a significant predictor of adverse pregnancy outcomes. The area under the curve was calculated as 0.659 [95% confidence interval (CI): (0.562-0.756); p=0.001] (Figure 2).

## DISCUSSION

Although no single screening test in the prediction of adverse pregnancy outcomes, especially preeclampsia, has gained widespread adoption into clinical practice, uterine artery Doppler screening is the best performing of the available clinical tests to date, and is certainly the most widely studied. The association between increased uterine artery PI and subsequent development of preeclampsia is thought to be the consequence of impaired trophoblastic invasion of maternal spiral arteries^(7)^. Preeclampsia, which is genetically and immunologically governed, constitutes a disease of circulatory maladaptation to this defective trophoblastic invasion. In normal pregnancy, the luminal diameter of spiral arteries is greatly increased and vascular smooth muscle is replaced by trophoblast cells. In preeclampsia, the process is deficient with a consequent decrease in the vascular capacitance and increased resistance in the uteroplacental circulation, which is reflected as impedance of blood flow in the uterine artery^(8)^. FGR is also the result of impaired blood flow to the patient.

Abnormal second-trimester uterine artery Doppler indices have a high detection rate of pregnancies at risk of preeclampsia and FGR. In this study, 40.6% of patients with abnormal uterine artery Doppler developed preeclampsia.

FGR was found in 31.2% patients with abnormal uterine artery Doppler. A percentage (31.2%) of the patients also had small-for-gestational-age (SGA) babies.

Albaiges et al.^(9)^ reported similar findings, with a higher detection rate for preeclampsia and SGA. Our study also reported that second-trimester uterine artery Doppler had high negative predictive value for preeclampsia. The sensitivity for preeclampsia was 61.9% and low birth weight was 45.4%, compared with 40% and 20%, respectively, in the study by Albaiges et al.^(9)^ In a separate study by Harrington et al.^(10)^ 81.2% of patients with abnormal uterine artery Doppler developed preeclampsia and 57.6% of these patients had low-birth-weight babies.

In our study, the positive predictive value of abnormal second-trimester uterine artery Doppler in determining at least one adverse outcome was 84.4%, which was significant. The likelihood ratio, sensitivity and specificity for each Doppler index and specific outcome varied among previous studies, but the predictive relationship for adverse outcomes has been consistently reported.

Our study found no association between deranged second–trimester uterine artery Doppler and spontaneous preterm delivery (delivery <37 weeks). This was in contradiction to the study by Fonseca et al.^(11)^ in 2006, which demonstrated a significant association of mean bilateral increased uterine artery PI at 22-24 weeks and spontaneous early delivery. However, the study also concluded that uterine artery Doppler does not provide a significant improvement in the prediction of spontaneous early delivery provided by maternal characteristics and previous obstetric history.

Patients with both elevated PI and bilateral notching in the Doppler were found to be at the maximum risk of adverse pregnancy outcomes, especially preeclampsia and FGR. Albaiges et al.^(9)^ reported the risk of preeclampsia as 40%, and 45% for FGR in these patients. These findings suggest that patients with elevated PI and notching at 23 weeks should be closely monitored for these adverse outcomes.

Our study concludes that second-trimester uterine artery Doppler has a potential role in predicting pregnancies at risk for complications such as preeclampsia, FGR, and small-for-gestational-age babies. This study also noted the high negative predictive value of uterine artery Doppler for adverse perinatal events among unselected women. It signifies that pregnancy outcome is likely to be normal if second-trimester uterine artery Doppler is normal. Although there is no effective intervention at present to alter outcomes in women with an abnormal Doppler study, the level of antenatal surveillance could be modified by the Doppler result^(12)^. However, uterine artery Doppler alone for predicting pregnancy outcomes has a limiting factor of cost effectiveness. A large number of Doppler studies have to be performed to identify a few high-risk women, which may not be cost effective. It is not logistically feasible in regard to availability and expertise of personnel to perform uterine artery Doppler in all pregnant women.

The strength of this study is its prospective nature, albeit with a small sample size and small number of patients with abnormal second-trimester uterine artery Doppler. Larger randomized controlled trials are required for second-trimester uterine artery Doppler for the extrapolation of the results to the whole population.

## Figures and Tables

**Table 1:**
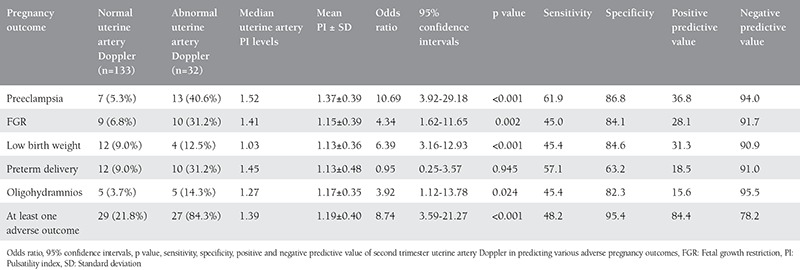
Normal and abnormal second-trimester uterine artery Doppler in adverse pregnancy outcomes and median uterine artery Doppler pulsatility index value

## References

[1] Bahado-Singh RO, Jodicke C (2010). Uterine artery Doppler and first trimester screening. Clin Obstet Gynecol.

[2] Melchiorre K, Wormald B, Leslie K, Bhide A, Thilaganathan B (2008). First-trimester uterine artery Doppler indices in term and preterm pre-eclampsia. Ultrasound Obstet Gynecol.

[3] Cnossen JS, Morris RK, ter Riet G, Mol BW, et al (2008). Use of uterine artery Doppler ultrasonography to predict pre-eclampsia and intrauterine growth restriction: a systematic review and bivariable meta-analysis. CMAJ.

[4] Costa SL, Proctor L, Dodd JM, Toal M, Okun N, Johnson JA, et al (2008). Screening for placental insufficiency in high risk pregnancies: is earlier better?. Placenta.

[5] Chien PF, Arnott N, Gordon A, Owen P, Khan KS (2000). How useful is uterine artery Doppler flow velocimetry in the prediction of preeclampsia, intrauterine growth retardation and perinatal death? An overview. BJOG.

[6] Ducey J (1989). Velocity waveforms in hypertensive disease. Clin Obstet Gynecol.

[7] Spencer K, Yu CK, Cowans NJ, Otigbah C, Nicolaides KH (2005). Prediction of pregnancy complications by first trimester maternal serum PAPP-A and free β-hCG and with second trimester uterine artery Doppler. Prenat Diagn.

[8] Wilson ML, Goodwin TM, Pan VL, Ingles SA (2002). Molecular epidemiology of preeclampsia. Obstet Gynecol Surv.

[9] Albaiges G, Missfelder-Lobos H, Lees C, Parra M, Nicolaides KH (2000). One-stage screening for pregnancy complications by colour Doppler assessment of the uterine arteries at 23 weeks gestation. Obstet Gynecol.

[10] Harrington K, Cooper D, Lees C, Hecher K, Campbell S (1996). Doppler ultrasound of the uterine arteries: the importance of bilateral notching in the prediction of pre-eclampsia, placental abruption or delivery of a small-for-gestational-age baby. Ultrasound Obstet Gynecol.

[11] Fonseca E, Yu CK, Singh M, Papageorghiou AT, Nicolaides KH (2006). Relationship between second-trimester uterine artery Doppler and spontaneous early preterm delivery. Ultrasound Obstet Gynecol.

[12] Bower SJ, Harrington KF, Schuchter K, MacGirr C, Campbell S (1996). Prediction of pre-eclampsia by abnormal uterine Doppler ultrasound and modification by aspirin. Br J Obstet Gynecol.

